# Quadruple fenestration aortic stent implantation combined with unilateral IBE and internal iliac artery stent implantation for complex abdominal aortic aneurysm: one case report

**DOI:** 10.3389/fcvm.2023.1276064

**Published:** 2023-10-10

**Authors:** Guanzhi Zhou, Huibo Ma, Junjun Liu, Xiaozhi Sun, Yangshuo Liu, Jianli Luan, Yongxin Li, Mingjin Guo

**Affiliations:** Department of Vascular Surgery, The Affiliated Hospital of Qingdao University, Qingdao, China

**Keywords:** endovascular aneurysm repair, fenestration technology, iliac branch endoprosthesis, complex abdominal aortic aneurysm, iliac aneurysm

## Abstract

An abdominal aortic aneurysm is a frequently encountered clinical condition, which necessitates prompt and effective remediation to avoid rupture. Surgeons must meticulously select an appropriate method of repair and assess the long-term surgical prognosis when dealing with patients with complex abdominal aortic aneurysms. In this case report, a 74-year-old man was hospitalized due to acute abdominal pain. Upon further examination, it was discovered that he was suffering from a complex abdominal aortic aneurysm. The thoracoabdominal aorta CTA showed that the aneurysm involved both renal arteries, the part below the kidney was severely twisted, the neck of the aneurysm was short, and it was accompanied by bilateral common iliac and internal iliac aneurysms, and there were considerable thrombus attached to the vessel wall. In this case, our team used 3D technology to simulate the spatial structure of the aneurysm and comprehensively evaluate the patient's condition. Ultimately, we decided to perform a quadruple fenestration aortic stent implantation and endovascular repair of aortic aneurysm, combined with right IBE and internal iliac artery stent implantation, right internal iliac artery reconstruction, and left internal iliac artery aneurysm embolization on this patient. This is an innovative surgical method. The operation was successful and the patient recovered well after the operation.

## Introduction

In this article, we report the case of a 74-year-old male with a complex abdominal aortic aneurysm with extensive involvement. For this patient, our team decided to perform a four-window endovascular repair of the abdominal aortic aneurysm and bilateral iliac artery aneurysm endovascular isolation and right internal iliac artery reconstruction using The GORE EXCLUDER Iliac Branch Endoprosthesis (IBE), based on his condition and examination results, to reduce contraindications and complications. For this patient, our team decided to perform a quadruple fenestration aortic stent implantation and endovascular repair of aortic aneurysm, combined with right IBE and internal iliac artery stent implantation, right internal iliac artery reconstruction, and left internal iliac artery aneurysm embolization, based on his condition and examination results, to reduce contraindications and complications. Up to now, the patient has been followed up for 4 months, is in good general condition, and has resumed normal physical activity. This operation achieved successful clinical results.

## Case report

**Figure 1:**
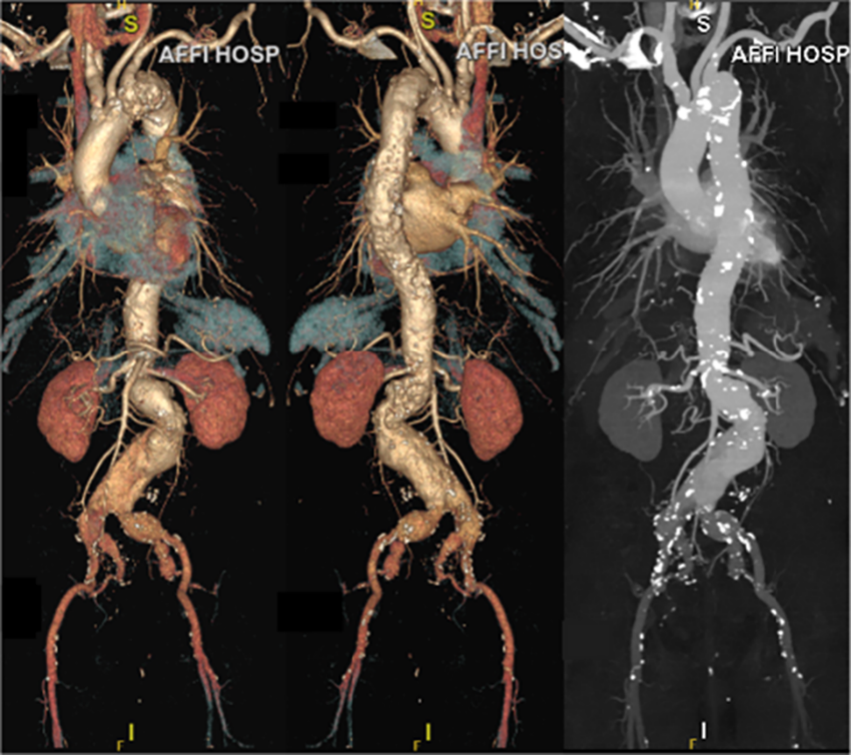
Preoperative CTA.

A 74-year-old man was admitted to the emergency department due to sudden severe abdominal pain lasting for about one hour. An immediate ultrasound examination of the patient's abdominal vessels revealed a large and complex abdominal aortic aneurysm. The patient had a history of hypertension for over 20 years, with a maximum blood pressure reaching 200/100 mmHg. He was taking oral medication to control his blood pressure, but the control was not effective. Upon physical examination, we detected a pulsatile mass in his lower abdomen measuring 7 cm by 6 cm. Following admission, the patient underwent comprehensive auxiliary examinations. The thoracoabdominal aorta CTA of the patient showed that there was an uneven widening from his aortic arch to the thoracoabdominal aorta to both common iliac arteries and to both internal iliac arteries. His aneurysm was mainly located at the lower end of the abdominal aorta, and the diameter of the widest part of the aneurysm was about 75 mm. At the same time, it revealed that the patient had a penetrating ulcer in the proximal aortic arch with severe calcification (Figure 1). In order to reduce the patient's risk of aortic arch disease, we gave the patient supportive treatments such as blood pressure control and anticoagulation during the perioperative period. After measuring and analyzing the imaging data, we found: the patient's abdominal aortic aneurysm involves bilateral renal arteries; the patient's infra-renal abdominal aorta is severely twisted and the aneurysm neck is very short, while the diameter of the supra-renal abdominal aorta is only 22 millimeters and there are changes of atherosclerosis internally; the patient has bilateral common iliac artery aneurysms and bilateral internal iliac artery aneurysms, with the left side being more prominent, while the middle and distal part of the right internal iliac artery is not dilated; the patient's internal iliac artery is twisted, accompanied by local stenosis and dilation. Through the analysis of the patient's imaging data and considering the patient's general physical condition, we finally decided on the surgical plan for the patient: quadruple fenestration aortic stent implantation and endovascular repair of aortic aneurysm, combined with right IBE and internal iliac artery stent implantation, right internal iliac artery reconstruction, and left internal iliac artery aneurysm embolization.

**Figure 2:**
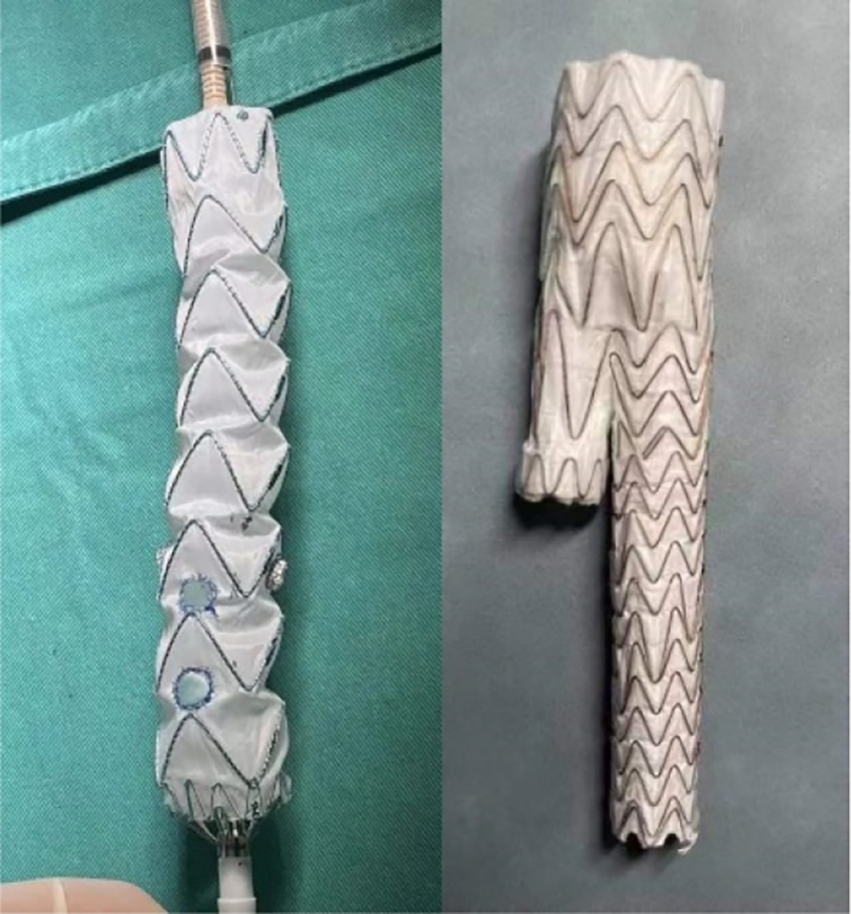
Bracket appearance (fenestration bracket and GORE EXCLUDER iliac branch endoprosthesis).

**Figure 3:**
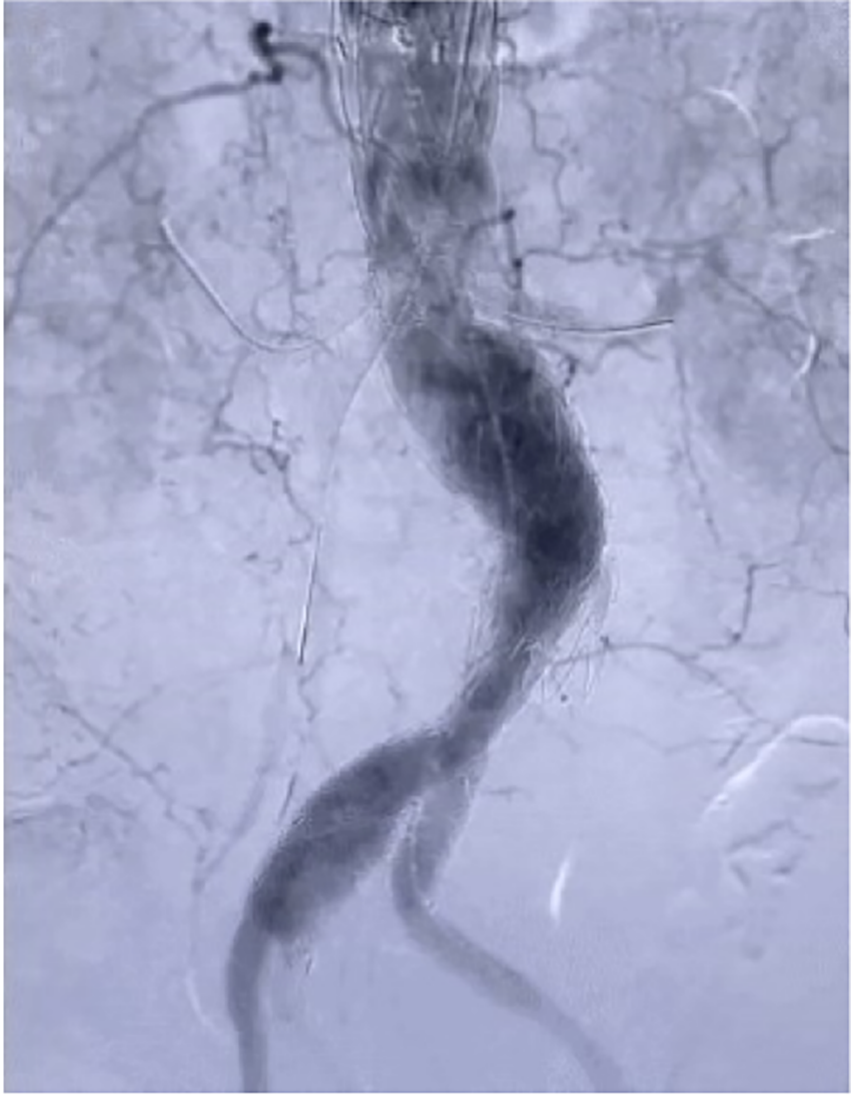
Postoperative angiography.

On August 17, 2022, after comprehensive preparation, the patient underwent surgery (Figure 2). The specific surgical process is as follows: First, we released the aortic stent graft *in vitro*, and based on preoperative measurements, we performed fenestration at the corresponding position of the stent and sutured the branch stent (Medtronic 3,632). Then, we placed the pre-prepared aortic stent graft along. Subsequently, we performed angiography on the patient to locate the positions of the bilateral renal arteries, the celiac artery, and the superior mesenteric artery. Following this, we placed the pre-prepared aortic stent graft along the guide wire to the appropriate position, performed angiography again, and confirmed that the openings of the bilateral renal arteries, the celiac trunk, and the superior mesenteric artery were aligned with the fenestration on the stent. Afterwards, we placed a balloon-expandable stent graft (Lifestream 8 mm 26 mm) in the left renal artery, a balloon-expandable stent graft (Lifestream 8 mm 37 mm) in the right renal artery, and a VBX balloon-expandable stent graft (8 mm × 39 mm) in the superior mesenteric artery. Once completed, we performed angiography again to confirm the patency of the bilateral renal arteries, the celiac trunk, and the superior mesenteric artery. Next, we embolized the left internal iliac artery by placing a embolization coil (Interlock 20 mm × 40 cm) posterior to it. We then placed the IBE main body (CEB23-12-10) stent along the right guidewire. Subsequently, we successively placed a Gore covered iliac branch stent (HGB16-10-07) and a VBX balloon-expandable stent graft (10 mm × 59 mm) inside the distal part of the internal iliac artery, and then released the IBE main body stent. Then we placed a Gore abdominal aortic stent graft (RLT35-14-14) along the guidewire. When its upper end overlapped with the lower end of the previously placed thoracic aortic stent, we released it until the contralateral branch opened. We then placed a PLC2710 iliac branch stent along the guidewire on the right and connected it to the IBE main body stent. Then we completely released the ipsilateral branch of the IBE main body stent, then placed the PLC1212 iliac branch stent and covered the opening of the left internal iliac artery. Finally, we performed angiography again. Finally, we performed angiography again. The results showed that the abdominal aorta, bilateral renal arteries, celiac trunk, superior mesenteric artery, bilateral common iliac arteries, bilateral external iliac arteries, and right internal iliac artery were all unobstructed, and the aneurysm isolation effect was good (Figure 3).

**Figure 4:**
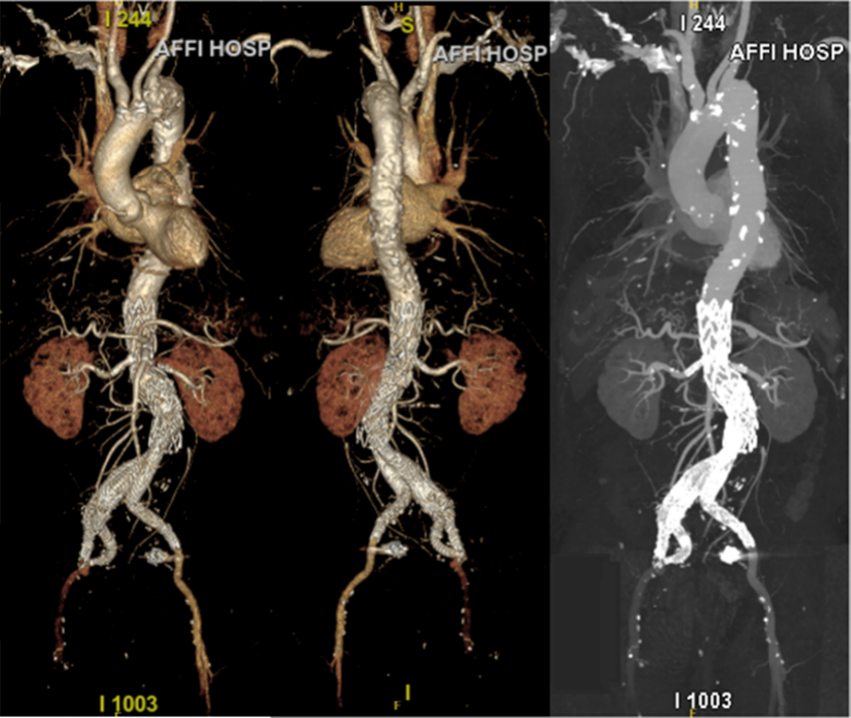
Postoperative CTA.

After the operation, we administered antibiotics to the patient to prevent infection and used low molecular weight heparin for anticoagulation treatment, all while closely monitoring the patient's vital signs. One week after the surgery, we again conducted a CTA examination of the patient's abdominal aorta. The results suggested a successful surgical outcome (Figure 4), thus we approved the patient's discharge. Four months after the surgery, we followed up with the patient. The patient was in generally good health, with no obvious discomfort, and had resumed normal activities.

## Discussion

Abdominal Aortic Aneurysm (AAA) is a common arterial dilation disease, characterized by localized, permanent dilation of the patient's abdominal aortic wall, with a diameter more than 50% larger or exceeding 3 cm compared to the adjacent normal abdominal aorta. Complex Abdominal Aortic Aneurysms (cAAA) generally refer to abdominal aortic aneurysms with a short aneurysm neck or those that involve the visceral arteries (1). Currently, in clinical practice, surgeons mainly treat patients with moderate to high-risk AAA through surgery. There are two surgical treatment options for AAA: open surgery and Endovascular Aneurysm Repair (EVAR). In the past, the treatment for abdominal aortic aneurysms mainly involved open surgery, which consisted of using artificial vascular grafts to replace the affected parts of the abdominal aorta. The advantage of open surgery is that it's more direct and the therapeutic effect is more definitive (2). However, traditional open surgery has several drawbacks, including large surgical incisions, long operation time, disruption of other organs in the abdominal cavity, and an increased risk of various perioperative complications. EVAR refers to an interventional treatment method that transports and places the covered stent component through the arterial route into the aneurysm, thereby isolating the aneurysm cavity from the bloodstream, allowing blood to flow to the distal end through the covered stent. Research shows that compared with traditional surgery, EVAR in the treatment of AAA patients has advantages such as being less invasive, shorter hospitalization times, quicker postoperative recovery, and a lower occurrence rate of complications. Concurrently, for AAA with subpar anatomical structure, EVAR can achieve good outcomes. Nevertheless, for overly complex AAA, there are certain limitations to the application of EVAR. The patient is elderly, has a history of hypertension and poor blood pressure control, is at risk of aortic arch disease, and is in poor general health. Therefore, EVAR is a better choice than open surgery for this patient. Because although EVAR is more difficult to deal with such a complex aneurysm, it can effectively reduce the trauma of surgery to the patient, facilitate postoperative recovery, and reduce the occurrence of postoperative complications.

The main difficulty in treating cAAA are manifested in adequately assessing the situation of the aneurysm's proximal anchoring area and the rational reconstruction of the visceral branch arteries around the aneurysm (3). The abdominal aortic aneurysm stent graft fenestration endovascular repair is an intracavitary technique used to reconstruct the blood supply of the visceral branch arteries surrounding the abdominal aortic aneurysm. It can reduce the risk of type Ia endoleaks while preserving the blood supply to the important branch arteries of the aorta (4). However, this technique requires ensuring the anastomosis of the branch arteries with the fenestration on the aortic stent during the operation. Therefore, it is difficult to use fenestration stent technology for severely curved visceral arteries. In this case, the AAA suffered by the patient involved both renal arteries, the celiac artery, and the superior mesenteric artery. In order to ensure the blood supply to the patient's vital organs, we chose to use precise extracorporeal fenestration technology to protect the branch arteries. However, the patient's abdominal aortic aneurysm morphology was quite twisted, and it involved multiple visceral branch vessels, as many as four. Therefore, we encountered certain challenges in the design of the overall structure of the stent and the selection of specific locations.

According to statistics, about 43% of abdominal aortic aneurysm patients have lesions involving one side of the common iliac artery, and about 11% involve both sides of the common iliac artery (5, 6). Involvement of the internal iliac artery is a common phenomenon in abdominal aortic aneurysm combined with common iliac artery aneurysm. In conventional EVAR, to prevent type Ib endoleaks, the surgeon usually needs to extend the stent to the level of the external iliac artery. At the same time, to avoid type II endoleaks caused by reflux in the internal iliac artery, surgeons often have to embolize the internal iliac artery. However, the pelvic ischemia caused by embolism of the internal iliac artery may lead to a series of complications, among which claudication of the gluteal muscles and sexual dysfunction are more common, accounting for about 28% and 17%, respectively. Severe cases can even cause paralysis and other symptoms such as intestinal ischemia (7). The severity of the condition depends on the patency of the internal iliac artery before occlusion and the compensation of the collateral circulation after occlusion, usually from the branches of the contralateral internal iliac artery and the ipsilateral and contralateral deep femoral artery (8, 9). Therefore, how to avoid endoleaks while preserving or reconstructing the internal iliac artery to ensure the quality of life of patients is a difficulty in the treatment of common iliac artery aneurysm. IBE technology is to use the iliac artery branch stent graft (IBE stent) to reconstruct the common iliac artery while separating the aneurysm cavity and reconstructing the internal iliac artery. In the reconstruction of the internal iliac artery, there were some deficiencies in the past parallel stent technology and fenestration technology, but the branch design can better complete the reconstruction of the internal iliac artery. IBE can reduce complications and maintain the quality of life of patients by preserving blood flow in the internal and external iliac arteries. Moreover, the anchoring points of the branch stents have sufficient overlap, thus reducing the likelihood of endoleaks. In this case, the patient had an aneurysm combined with aneurysms of the bilateral common iliac arteries and bilateral internal iliac arteries, where the left side was more obvious, and no expansion was observed in the middle and distal sections of the right internal iliac artery. Therefore, after comprehensive consideration, we chose to embolize the left internal iliac artery aneurysm while using IBE technology to protect and reconstruct the right internal iliac artery, thereby reducing the occurrence of surgical complications.

For complex abdominal aortic aneurysms involving multiple visceral arteries and Type IV thoracoabdominal aortic aneurysms, most medical centers choose to handle them with “fenestration combined with branch stent technology” (10). Compared with simple stent fenestration technique, the fenestration combined with branch stent technique is more flexible and has lower requirements for the anatomical structure of the aneurysm. In this case, our team simulated the spatial structure of the patient's aneurysm using 3D spatial synthesis technology, according to the patient's condition, and precisely measured and calculated the related data. Subsequently, we repeatedly deliberated on the risks and problems that might occur during the operation, compared the advantages and disadvantages of different plans, and finally determined the surgical plan for this patient, that is, the quadruple fenestration aortic stent implantation and endovascular repair of aortic aneurysm, combined with right IBE and internal iliac artery stent implantation, right internal iliac artery reconstruction, and left internal iliac artery aneurysm embolization. Both of the techniques applied in this surgery are complex when used separately. However, considering the current condition of the patient, simultaneously using these two surgical techniques may be the best option, so we integrated both techniques into the same surgery for this patient. This kind of surgical plan that combines the two techniques is currently relatively rare in the existing literature. Although it makes the operation significantly more difficult, it is the solution with the best expected results for this patient, and it can also reduce the patient's surgical injuries and postoperative complications. This attempt is both a challenge and an innovation for us. The surgical plan resolved the abdominal aortic aneurysm and iliac artery aneurysm at once, while effectively isolating the aneurysm and preserving the blood supply to the branch arteries, thus reducing surgical complications and resolving the issues in a safe and efficient manner.

## Data Availability

The raw data supporting the conclusions of this article will be made available by the authors, without undue reservation.
